# A Highly Efficient and User‐Friendly Sensitive Skin Model on the Forearm

**DOI:** 10.1111/jocd.16619

**Published:** 2024-10-09

**Authors:** Jianhua Zhang, Shichao Liu, Wenjiao Guo, Na Li, Yun Huang

**Affiliations:** ^1^ N.O.D Topia (GuangZhou) Biotechnology Co., Ltd. Guangdong Guangzhou China; ^2^ Simpcare (GuangZhou) Biotechnology Co., Ltd. Guangdong Guangzhou China

**Keywords:** capsaicin test, efficacy evaluation, forearm, sensitive skin model, skin redness, tape stripping

## Abstract

**Background:**

Sensitive skin is a worldwide skin problem, and its assessment of therapeutic efficacy traditionally relies on the facial stinging test. However, this test possesses certain limitations due to its restrictive application site, intense pain sensation, and adverse effects on physical appearance.

**Objective:**

This study aimed to develop and evaluate a highly efficient and user‐friendly sensitive skin simulation model, which combines tape stripping and capsaicin testing on the forearm (FA‐TS‐CAT), as an alternative to the facial stinging test.

**Methods:**

A randomized, double‐blind, placebo‐controlled clinical trial was conducted involving 74 subjects. Skin redness (a* value), transepidermal water loss (TEWL), and self‐assessment questionnaires were collected at different time points for analysis.

**Results:**

Tape stripping 5 times and 10 min application of capsaicin test were identified as the optimal conditions for the FA‐TS‐CAT model. Consistent stimulation and natural recovery trends of a* value and TEWL were observed on both the FA‐TS‐CAT and facial capsaicin test (F‐CAT) models within 50 min. After the 4‐*t*‐butylcyclohexanol complex emulsion was applied, the a* value in the FA‐TS‐CAT model exhibited a soothing trend similar to the F‐CAT model, with a significantly reduced by 3.99‐fold and 3.28‐fold at T3 and T4 (*p* < 0.001), compared to the placebo. Notably, the test efficiency of the FA‐TS‐CAT model was threefold higher than that of the F‐CAT model, and subjects showed more willingness to participate in the FA‐TS‐CAT test (95.95% vs. 4.05%).

**Conclusions:**

These results indicated the FA‐TS‐CAT is a highly efficient and user‐friendly model for sensitive skin, providing a reliable and valid method for clinical research in sensitive skin treatment.

## 1 Introduction

Over 70% of the global population reports having some degree of sensitive skin (SS), with over 40% of these individuals experiencing moderate to high levels of skin sensitivity [1]. SS is defined by the self‐reported facial presence of unpleasant sensations, including tingling, burning, pain, and itching, in response to stimuli that should not provoke such sensations [2]. Although the pathophysiological mechanisms of SS are not yet clear, it is generally believed that neurosensory hyperactivity and impaired epidermal barrier function are two potential mechanisms associated with SS [3]. Previous studies have demonstrated an upregulation of transient receptor potential vanilloid‐1 (TRPV1) expression in individuals with SS, and this upregulation has been correlated with the intensity of symptom [4]. Higher sensitivity has been reported to be associated with barrier dysfunction, and a reduced number of corneocytes, rendering individuals more susceptible to chemical irritants [5]. Consequently, TRPV1 antagonists and barrier repair agents/products have been engineered to alleviate the discomfort experienced by patients with sensitive skin. To more efficiently screen potential treatments for SS, the development of a stable and safe skin model is essential.

The nasolabial fold stinging test, utilizing irritants like capsaicin (CAT) or lactic acid (LAST), has been widely employed as a diagnostic tool to distinguish between sensitive and non‐sensitive skin. It is also utilized to evaluate the soothing effects of skincare products and active ingredients [6]. However, the irritants may induce intense burning sensations on the face, extensive redness, and compromise the integrity of the skin barrier [7]. Therefore, considering the significance of the face in personal appearance, individuals with SS, may be unwilling to participate in CAT or LAST. Additionally, CAT and LAST are inefficient as they are confined to comparing the split‐face between two samples. Since SS is a subjective condition, the stinging tests are often combined with subjective assessment scales. Therefore, a self‐assessment questionnaire that collected and scored the various discomforts experienced by the subject may be the most appropriate method of assessing the efficacy of treatment for SS. Recently, some researchers have developed in vitro stinging models using skin cells or 3D epidermal models to achieve efficient clinical screening of cosmetics. However, these single‐cell cultures overlook the critical cell‐to‐cell communication found in human skin [8]. Meanwhile, these 3D models have limitations in evaluating improvements in neural sensitization, a key regulatory mechanism in SS [9]. The models do not fully address the combined needs for efficiency, safety, and an objective reflection of actual human skin conditions and subjective perception.

Therefore, we proposed the idea of transferring the facial stinging test to other parts of the body. The forearm and back are commonly used locations for skin testing besides the face [10]. Unlike the nasolabial fold, the forearm provides a larger test area with a lower impact on appearance. Compared to the back, the forearm has a thinner stratum corneum, which facilitates experimental procedures for researchers and is more friendly for subjects. Slight tape stripping can make the forearm's stratum corneum more similar to that of facial skin. This technique, although “destructive,” is well‐tolerated by subjects [11]. It also enhances capsaicin penetration to imitate the skin conditions in facial stinging tests on SS. Based on this premise, a highly efficient and user‐friendly sensitive skin test model, which combines tape stripping and capsaicin testing on the forearm (FA‐TS‐CAP) was developed. Subsequently, the consistency and substitutability between the FA‐TS‐CAP model and the facial capsaicin test (F‐CAT) model with and without the use of soothing products were evaluated.

## 2 Methods

### 2.1 Materials

Capsaicin (purity > 98%) was purchased from Beijing Solarbio Science & Technology Co., Ltd. (Beijing, China), and 90% ethanol and filter paper were purchased from Shanghai Maclin Biochemical Technology Co., Ltd. (Shanghai, China). Medical tape (2.4 cm × 9.8 m) was purchased from 3M Company. 4‐*t*‐butylcyclohexanol was provided by Symrise AG, Germany (INCI: 4‐*t*‐butylcyclohexanol, 1,2‐pentanediol). *Crocus sativus* flower extract was provided by SEQENS, France (INCI: water, 1,3‐propylene glycol, *Crocus sativus* flower extract), and *Gentiana rigescens* extract was obtained from Yunnan Ingredi Biological Co., Ltd., Yunnan, China (INCI: *Gentiana rigescens* extract, maltodextrin).

### 2.2 Subjects

This randomized, double‐blind clinical trial was carried out on 74 healthy subjects aged 20–50 years, comprising 67 facial capsaicin‐positive subjects. Facial capsaicin‐positive subjects participated in the F‐CAT model and forearm model. The F‐CAT was slightly modified based on the method developed by Hu [12]. A two‐layer filter paper with a diameter of 0.8 cm containing capsaicin aqueous solution (0.01% w/v, 50 μL) was placed on both sides of the nasolabial fold and waited for 2 min. A 1–5 semi‐subjective rating scale [12] to assess the intensity of pain was employed (1, doubtful, barely perceptible; 2, slightly perceptible; 3, moderate perceptible; 4, strong perceptible; 5, pain). Subjects with a pain sensation in capsaicin‐treated area ≥ 3 points and a duration > 30 s were considered positive for the capsaicin test.

Subjects with chronic or acute dermatitis in the test area who used topical or oral immunosuppressive drugs in the past month were excluded. Cosmetics were not allowed to be applied to the testing area within 3 days prior to the start of the study. Additionally, subjects were instructed to avoid activities such as sun lounging, sauna use, swimming, and strenuous physical exertion. This study was conducted in accordance with the Helsinki Declaration and the ICH GCP guidelines as applicable to cosmetic products. The study protocol was approved by the Human Research Ethics Committee (SLLS2023003). All subjects were kept informed of all risks associated with the experiments before signing the informed consent form, and all participated voluntarily in this study. The subjects have agreed to publish the pictures involved in the paper.

All instruments were operated by trained and experienced testers. Before measurements, all subjects washed their faces and forearms. The experiments were conducted after the subjects acclimated to a controlled environment (room temperature at 21°C ± 1°C, relative humidity of 50% ± 10%) for at least 30 min.

### 2.3 Establishment of the FA‐TS‐CAT Model

#### 2.3.1 Optimization of the Conditions for the FA‐TS‐CAT Model

**FIGURE 1:**
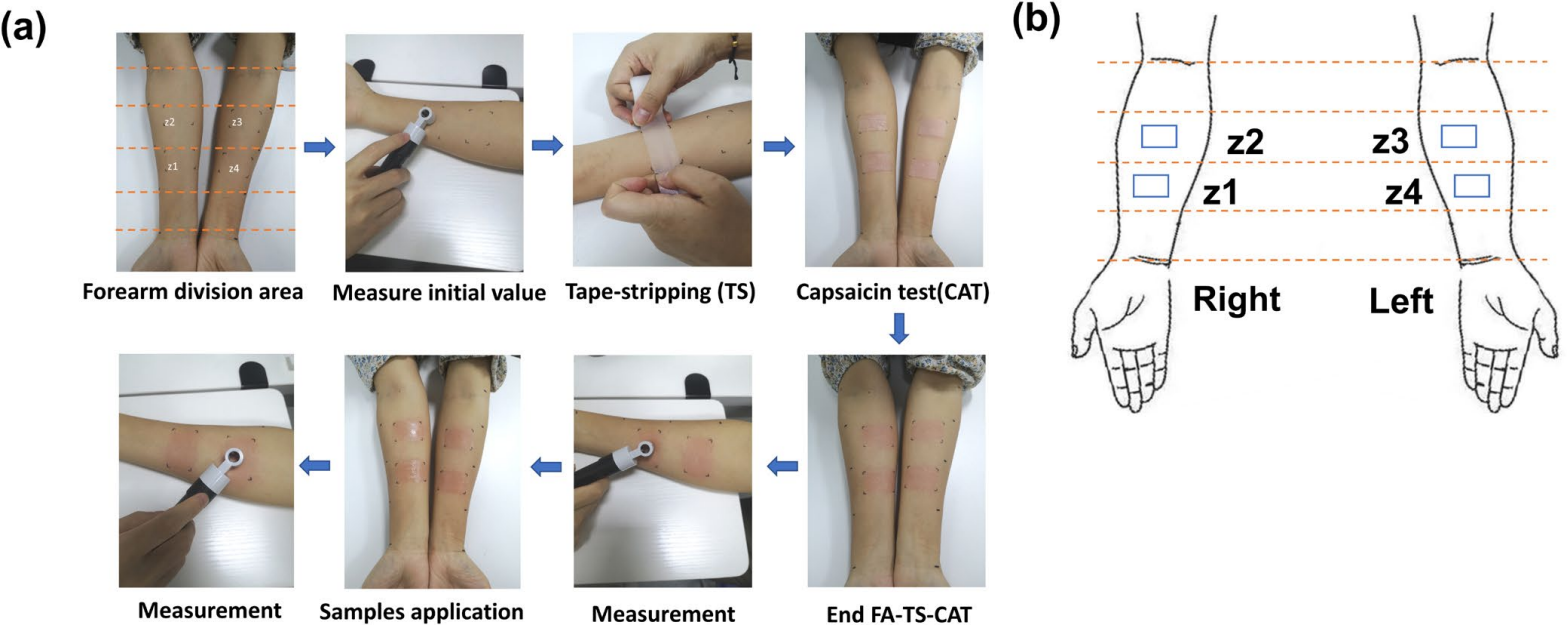
Establishment of the FA‐TS‐CAT model. (a) The experimental process of the FA‐TS‐CAT model. (b) Schematic of the forearm experimental areas.

Seventy‐two subjects were randomly divided into one of nine groups. The inner forearms were uniformly divided into four parts, avoiding areas with dense blood vessels. Four 2.5 cm × 4 cm (10 cm^2^) zones in the middle two parts, labeled as Z1, Z2, Z3, and Z4 (Figure 1), were selected for measurement. The skin redness (a* value) and transepidermal water loss (TEWL) were measured using the Colorimeter CL400 and TewaMeter TM Hex (MPA, Courage‐Khazaka Electronic GmbH, Koln, Germany) before any treatment (T0).

To determine the optimal experimental conditions for the FA‐TS‐CAT model, a three‐factor and three‐level L_9_(3^3^) orthogonal array table was designed, considering the stripping cycles with 3M tape (5, 10, and 15 times), application duration of CAT (5, 8, and 10 min), and measurement time (5, 10, and 15 min) of instruments (Table 1). After applying 3M tape stripping, non‐woven double layers measuring 2.5 cm × 4 cm (10 cm^2^) patches were soaked in a diluted 0.01% (w/v) capsaicin aqueous solution and applied to the test area for various durations. The a* values and TEWL were measured again and recorded as T0'.

**TABLE 1:** Control factors and selected levels of L9(3^3^) orthogonal array.

Factors	Level 1	Level 2	Level 3
Stripping cycles (×)	5	10	15
Application duration (min)	5	8	10
Measurement time (min)	5	10	15

The change rate for the a* value and TEWL in each area was calculated using the formula: change rate = (measured value—initial value)/initial value × 100%. The change rate of the a* value and TEWL in different test areas was analyzed to assess consistency. Subjects also provided self‐assessments of pain and willingness to participate in the test through a questionnaire. The significance levels of the three orthogonal factors were evaluated based on the range (R) and variance analysis. The K value and R value were calculated using Deming Chen's method [13]. The factor with the largest R value exerted the most significant influence on the change rate. The specific significance levels were indicated by the significance of the variance.

#### 2.3.2 Comparison Analysis of Skin Responses After FA‐TS‐CAT and F‐CAT Models

Utilizing the conditions of the FA‐TS‐CAT model optimized by the orthogonal experiment, 33 facial capsaicin‐positive subjects were randomly divided into three groups. Both the F‐CAT and FA‐TS‐CAT tests were performed simultaneously. In the F‐CAT test, a diameter of 0.8 cm 0.01% capsaicin patch was placed on both sides of the nasolabial fold until obvious redness was observed. In the FA‐TS‐CAT test, after tape stripping the skin a corresponding number of times, patches impregnated with 0.01% capsaicin were placed on Z1, Z2, Z3, and Z4. The a* value and TEWL measurements were collected at 10, 20, 30, 40, and 50 min post‐patch removal, denoted as T0', T1, T2, T3, and T4, respectively. The rate of change in the a* value and TEWL at T0' (model establishment) was calculated as follows: change rate = (measured values—initial value)/initial value × 100%.

Concurrently, subjects were administered a questionnaire at 6‐time points (T0', Ti—the time of capsaicin patch removal, T1, T2, T3, and T4) employing a 1–5 semi‐subjective rating scale [12] to assess intensity of pain.

#### 2.3.3 Evaluation of a Separate Forearm TS or CAT Test

Twenty‐two subjects were enlisted to investigate the necessity of combining tape stripping (TS) and capsaicin testing (CAT). Subjects were randomly conducted either 5 times of TS or 10 min of CAT at one of four forearm test areas, and no interventions were applied as a blank control. The a* values and TEWL were measured at T0, T0', T1, T2, T3, and T4.

### 2.4 Analysis of Therapeutic Efficacy of the 4‐*t*‐Butylcyclohexanol Complex on FA‐TS‐CAT and F‐CAT Models

#### 2.4.1 Samples

The test emulsion utilized in this study containing 2 wt% 4‐*t*‐butylcyclohexanol complex (4‐*t*‐butylcyclohexanol, *Crocus sativus* flower extract, and *Gentiana rigescens* extract combination at 1:0.5:0.5). The placebo is the same emulsion without the 4‐*t*‐butylcyclohexanol complex. The acute skin irritation test and skin patch test were conducted to confirm the excellent skin compatibility of the formulated test emulsion.

#### 2.4.2 Evaluation of the Efficacy of the 4‐*t*‐Butylcyclohexanol Complex Emulsion After the FA‐TS‐CAT and F‐CAT Models

A total of 34 facial capsaicin‐positive subjects were included in this efficacy test of 4‐*t*‐butylcyclohexanol complex emulsion. Two test areas were randomly selected from four test zones on the forearm. The initial a* value and TEWL of both the face and forearm were measured before the test (T0). The F‐CAT and FA‐TS‐CAT models were performed simultaneously, and the a* value and TEWL were measured after 10 min (T0'). The rate of change between T0 and T0' was calculated. The sample (200 μL) was randomly applied to one side of the nasolabial fold and forearm, while the placebo (200 μL) was applied to the remaining side. Measurements of the a* value and TEWL of the test areas were taken at T1, T2, T3, and T4. Facial and forearm images were captured by VISIA 7 (Canfeld Imaging Systems, Fairfeld, NJ, USA) and TiVi 700 (WheelsBridge AB, Sweden). The efficacy of the two emulsions to soothe irritation and improve redness was represented by the improvement rate in the a* value and TEWL, as follows: improvement rate = (measured values at each time point − T0' measurement value)/(T0' measurement value − T0 measurement value) × 100%.

#### 2.4.3 Self‐Assessment Questionnaire of Subjects

The questionnaire on a 1–5 semi‐subjective rating scale was employed to evaluate the intensity of pain experienced on the face and forearm at the time of CAT removal (Ti), 10 min after CAT removal (T0'), and at each subsequent time point following the use of sample or placebo.

### 2.5 Survey on the Experimental Willingness of Subjects

The sensations and willingness of all subjects who participated in the study were investigated, and their experiences were compared between the F‐CAT and FA‐TS‐CAT tests.

### 2.6 Statistics Analysis

The experimental data were statistically analyzed using SPSS 24.0 software and expressed as mean ± standard deviation (^−^x ± SD). A non‐parametric Mann–Whitney test was conducted to compare data between two groups. Intergroup differences were compared by one‐way analysis of variance (ANOVA), with significance levels indicated as follows: * *p* < 0.05, ** *p* < 0.01, and *** *p* < 0.001.

## 3 Result

### 3.1 Establishment of the FA‐TS‐CAT Model

#### 3.1.1 Optimization of the Conditions for the FA‐TS‐CAT Model

Based on the orthogonal design shown in Table 1, the change rate for the a* value and TEWL with different factors and levels was compared. The influence of three factors on change rate were ranked in descending order as follows: stripping cycles, application duration, and measurement time. The number of stripping cycles significantly impacted the change rate of the a* value and TEWL (Table 2). No significant difference was observed among Z1, Z2, Z3, and Z4 in the FA‐TS‐CAT test (Table 3). Therefore, we can infer that the number of stripping cycles plays a pivotal role in establishing the FA‐TS‐CAT model, and the experimental results are not limited by the testing area. Considering the limitation of the F‐CAT model to comparing the split‐face between two samples or a control sample and a test sample, we found that the testing efficiency of the FA‐TS‐CAT model was three times higher than that of the F‐CAT model.

**TABLE 2:** Orthogonal experimental results of the FA‐TS‐CAT model (mean ± SD).

Group no.	Stripping cycles	Application duration	Measurement time	Change rate of a* value (%)	Change rate of TEWL (%)
1	5	5	5	15.12 ± 2.54	15.58 ± 6.47
2	5	8	10	56.43 ± 6.21	22.72 ± 5.49
3	5	10	15	45.76 ± 3.92	15.18 ± 1.23
4	10	5	10	37.26 ± 6.21	21.36 ± 4.32
5	10	8	15	61.02 ± 3.27	53.27 ± 2.93
6	10	10	5	42.65 ± 4.93	26.61 ± 4.32
7	15	5	15	80.64 ± 2.31	58.09 ± 2.41
8	15	8	5	89.32 ± 2.08	73.62 ± 4.57
9	15	10	10	83.48 ± 2.32	59.87 ± 5.31
K1^a^	39.10	44.34	49.03		
K2^a^	46.98	68.92	59.06		
K3^a^	84.48	57.30	62.47		
R^a^	45.37	24.58	13.45		
*Significance* ^a^	0.02	0.54	0.83		
K1'^a^	17.92	31.77	38.69		
K2'^a^	33.75	49.87	34.65		
K3'^a^	63.86	33.89	42.18		
R'^a^	45.94	18.10	7.53		
*Significance*'^a^	0.07	0.62	0.93		

Abbreviations: K, the mean value of the index at each level of each factor; R, range.

^a^ K1, K2, K3, R and *significance* are the change rate of a* value, and K1', K2', K3', R and *significance* are the change rate of TEWL.

**TABLE 3:** Consistency analysis of the change rate of a* value and TEWL in the four zones on the left or right forearm.

	No.	Significance									
		T0	1	2	3	4	5	6	7	8	9
a* value	z1 versus Z2	0.12	0.66	0.87	0.74	0.96	0.66	0.15	0.64	0.78	0.90
	z1 versus Z3	0.07	0.83	0.93	0.82	0.73	0.89	0.72	0.86	0.97	0.59
	z1 versus Z4	0.74	0.77	0.98	0.91	0.72	0.88	0.96	0.61	0.88	0.97
	Z2 versus Z3	0.81	0.70	0.95	0.93	0.68	0.85	0.68	0.77	0.77	0.69
	Z2 versus Z4	0.11	0.79	0.91	0.87	0.67	0.84	0.32	0.99	0.92	0.87
	Z3 versus Z4	0.21	0.87	0.96	0.93	1.00	1.00	0.78	0.75	0.86	0.58
	L versus R	0.08	0.69	0.87	0.40	0.97	0.96	0.59	0.78	0.86	0.32
TEWL	z1 versus Z2	0.10	0.53	0.99	0.71	0.76	0.99	0.47	0.54	0.69	0.57
	z1 versus Z3	0.08	0.11	0.87	0.72	0.54	0.80	0.30	0.37	0.68	0.49
	z1 versus Z4	0.20	0.12	0.77	0.47	0.21	0.80	0.52	0.50	0.67	0.52
	Z2 versus Z3	0.92	0.08	0.88	0.98	0.78	0.81	0.76	0.81	0.95	0.71
	Z2 versus Z4	0.74	0.09	0.78	0.49	0.32	0.83	0.96	0.90	0.96	0.80
	Z3 versus Z4	0.66	0.39	0.89	0.55	0.38	0.65	0.83	0.95	0.99	0.89
	L versus R	0.14	0.39	0.92	0.39	0.78	0.70	0.51	0.59	0.75	0.67

*Note:* The a* values indicatethe significance analysis of the mean a* values or average change rates for the forearm’s four zones (Z1‐Z4) and the left and right hands across nine groups of subjects, both at baseline (T0) and after the construction of the FA‐TS‐CAT model.

Abbreviations: L, left forearm; R, right forearm.

**FIGURE 2:**
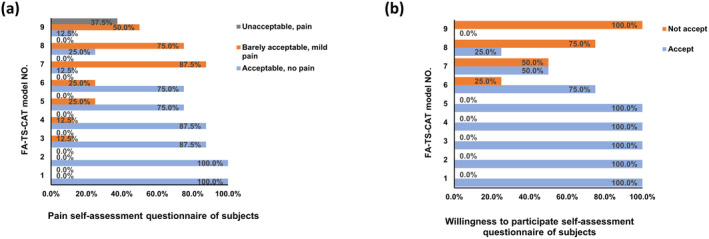
In the FA‐TS‐CAT model, the number of tape tripping cycles could not exceed 8, and the CAT application was limited to 10 min due to considerations of the subjects' pain (a) and willingness to participate (b).

The results of the questionnaire showed a substantial increase in the sensation of pain (Figure 2a) and a substantial decrease in willingness to participate (Figure 2b) when the number of stripping cycles reached 10 or the patching duration extended to 8 min. This trend was particularly pronounced after 15 stripping cycles and 10 min of patching, with the subjects expressing unbearable pain. FA‐TS‐CAT groups 2, 3, and 5 were selected for the subsequent tests based on the change rate of the a* value and TEWL, as well as the scores for pain sensation and willingness to participate. To mitigate the influence of the measurement time (T0'), measurements were conducted after 10 min.

#### 3.1.2 Comparison of Skin Responses After FA‐TS‐CAT and F‐CAT Tests

**FIGURE 3:**
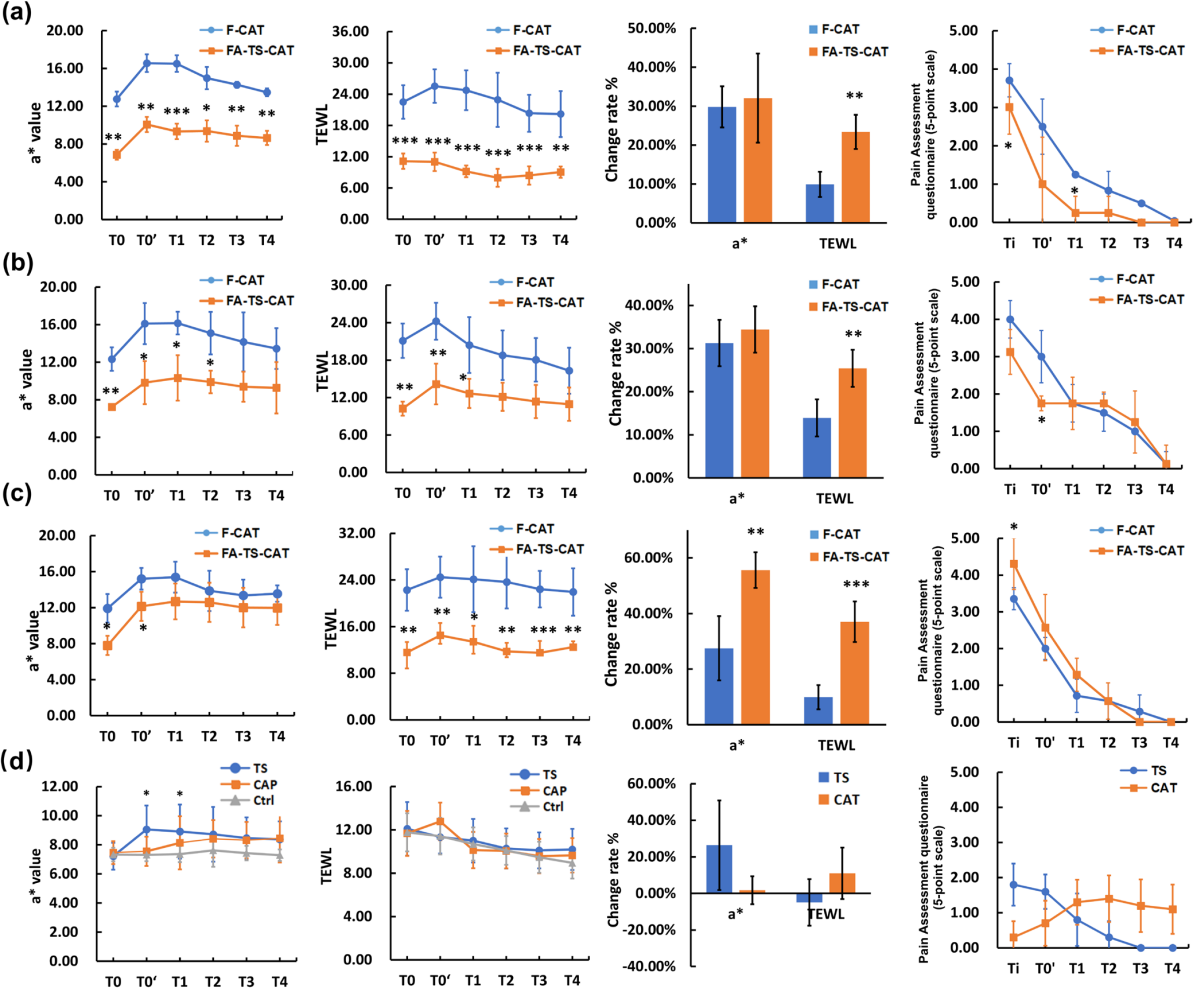
Natural skin recovery response in FA‐TS‐CAT model group 2 (a), 3 (b), and 5 (c). The trends in a* values, TEWL, change rates, and semi‐subjective pain scores for subjects in the FA‐TS‐CAT model group 3 (5 stripping cycles and 10‐min patch application) were consistent with those observed in the F‐CAT model (b). Tape‐stripping and capsaicin patches alone had no effect on the a* values or TEWL values on the forearm (d).

The changes in the a* value, TEWL, and semi‐subjective assessment of subjects showed the similar stimulation response and natural recovery trend in FA‐TS‐CAT group 3 (5 stripping cycles × capsaicin test application for 10 min) as in the F‐CAT model: the a* value and TEWL peaked at T1 and T0', followed by a subsequent decline (Figure 3). No significant difference was found in the rate of change in the a* value between FA‐TS‐CAT group 3 and the F‐CAT group. In conclusion, skin responses of FA‐TS‐CAT group 3 had the highest consistency with the F‐CAT test (Figure 3b).

#### 3.1.3 Separate the TS or CAT Application on the Forearm

The changes in a* value and TEWL following the application of TS or CAT on the forearm were shown in Figure 3d, and these results were inconsistent with the observed trend in the F‐CAT test. The semi‐subjective pain questionnaire responses from the subjects indicated that the rating for TS or CAT did not exceed 2 points, which is below the standard for facial capsaicin positivity. Therefore, the skin reaction after FA‐TS‐CAT was not solely caused by TS or CAT alone.

### 3.2 Evaluation of the Therapeutic Efficacy of the 4‐*t*‐Butylcyclohexanol Complex Emulsion

**FIGURE 4:**
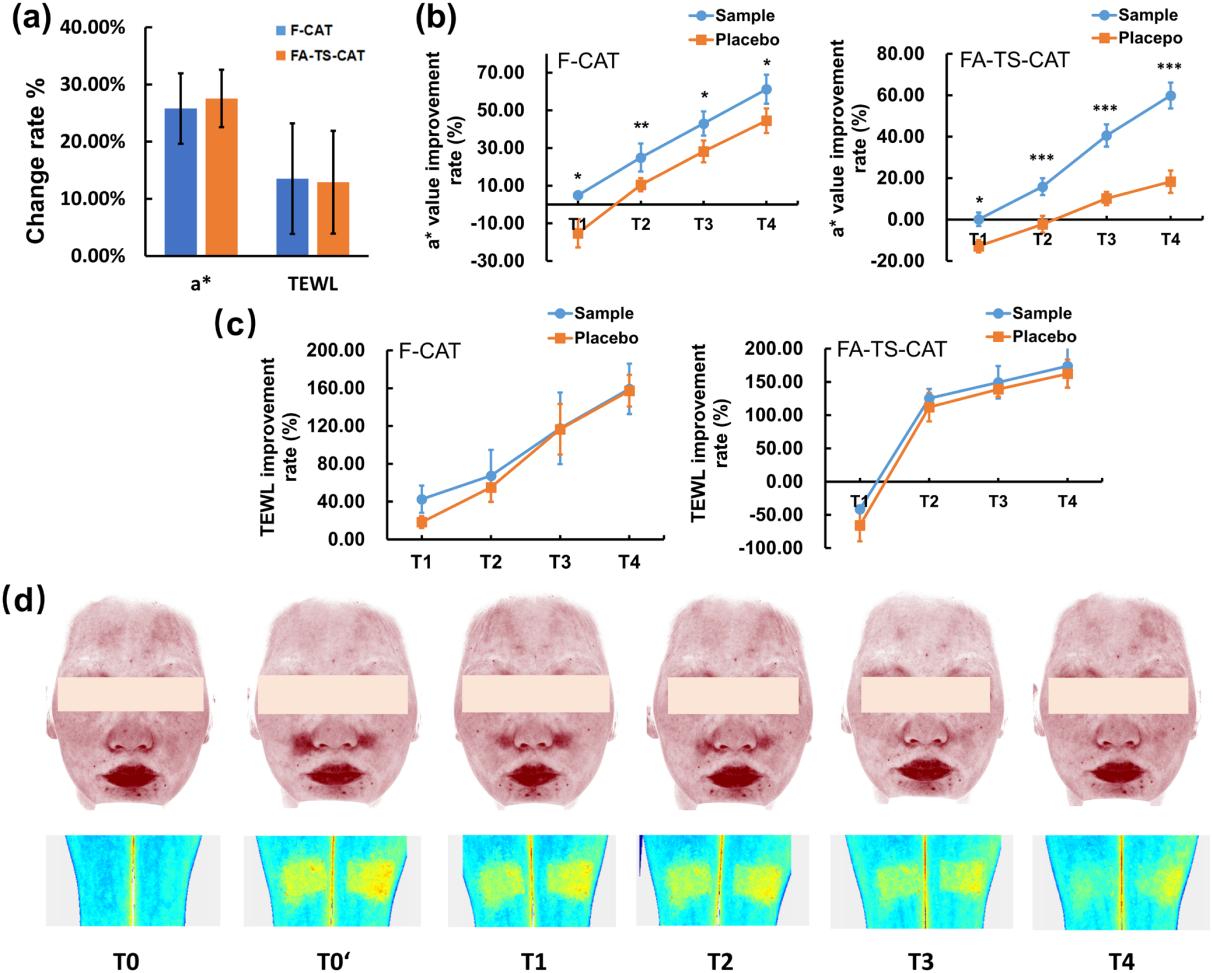
No significant differences were observed in the a* values and TEWL change rates between the FA‐TS‐CAT and F‐CAT models (a). The 4‐*t*‐butylcyclohexanol complex emulsion effectively reduced redness and soothes irritation on the face and forearms, demonstrating a consistent soothing trend (b, c). (d) Typical images of subject #30, sample was used for the left forearm and the left face, and placebo was used for the right forearm and the right face.

The change rates of the a* value and TEWL were presented in Figure 4a, showing no significant differences following the FA‐TS‐CAT and F‐CAT tests. Compared to the placebo, the a* value showed a significant decrease following the application of the sample on the face, with an improvement of 4.92% at T1 and 61.18% at T4, compared to −15.28% and 44.48% for the placebo (Figure 4b). After application of the sample on the forearm, the rate of improvement in a* values were 3.99 times that of the placebo at T3 and 3.28 times that of the placebo at T4 (Figure 4b). However, the improvement rate in TEWL displayed no significant differences between the sample and the placebo on both the face and forearm. These results demonstrated that the 4‐*t*‐butylcyclohexanol complex could relieve the stimulated reaction and improve redness (Figure 4d). The recovery trend of the FA‐TS‐CAT model after using the soothing product was consistent with the F‐CAT model.

### 3.3 Self‐Assessment Questionnaire of Subjects

**FIGURE 5:**
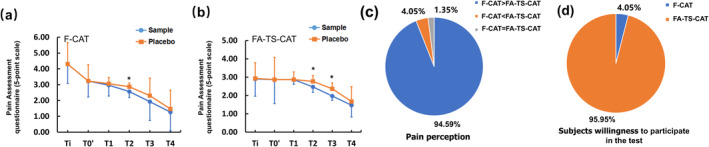
The efficacy of the sample in soothing the pain was stronger than that of the placebo both on the F‐CAT (a) and FA‐TS‐CAT (b) models. Subjects showed more willingness to participate in FA‐TS‐CAT than in F‐CAT (c, d).

In the F‐CAT model, the pain scores of the sample were significantly lower than those of the placebo at T2, with scores of 2.57 and 2.87, respectively (Figure 5a). Similarly, in the FA‐TS‐CAT model, the pain scores in the sample were significantly lower than the placebo at both T2 and T3 (Figure 5b). At the time point of Ti (immediately following the removal of the capsaicin test), the perception of pain caused by capsaicin on the forearm was significantly weaker compared to that on the face, with scores of 2.93 and 4.30, respectively.

### 3.4 Questionnaire of subjects' Willingness to Participate in F‐CAT or FA‐TS‐CAT Model

The questionnaires on pain and willingness to participate in these models of subjects were shown in Figure 5. 94.59% of the subjects reported experiencing stronger discomfort after the F‐CAT model (Figure 5c), and up to 95.95% were more willing to participate in the FA‐TS‐CAT model (Figure 5d). These findings strongly suggest the better adherence of subjects to the FA‐TS‐CAT test.

## 4 Discussion

Over the past two decades, with increasing global environmental pollution and individual psychological stress, more than half of the population has been identified as sensitive skin [1]. This skin problem has garnered widespread attention, prompting the development of assessment methods for skin sensitivity and efficacy assessment of soothing products [2]. Previous studies commonly utilized the facial stinging test, such as the CAT and the lactic acid stinging test (LAST), to identify sensitive skin. Subjects often expressed dissatisfaction and apprehension due to the resulting facial erythema and discomfort, particularly regarding changes in appearance [3]. Therefore, the safety of these models for assessment methods has a pressing need to be enhanced. To eliminate facial irritation, we established a standardized FA‐TS‐CAT model operating procedure (tape stripping 5 times and 10 min application of capsaicin test). The FA‐TS‐CAT model exhibited consistent skin response trends with the F‐CAT model, regardless of whether soothing products were applied. Additionally, the 4‐*t*‐butylcyclohexanol complex exhibited a good soothing effect in both the FA‐TS‐CAT and F‐CAT models. A greater number of subjects expressed a preference for participating in the FA‐TS‐CAT model rather than the F‐CAT model (Figure 5). The results showed that the FA‐TS‐CAT is an effective alternative to the traditional facial stinging test.

The hyper‐reactivity of nerves and blood vessels has been demonstrated as an important contributing factor to sensitive skin [14]. Research has indicated that the nasolabial fold, rich in nerves and blood vessels, is the most sensitive area on the face [15]. Numerous studies have identified sensitive skin and evaluated the soothing effects of treatments by observing the skin reactions to stimuli on the nasolabial fold [7, 8]. Nonetheless, persistent diffuse facial erythema and intolerable burning or pain sensations have been frequently reported as adverse effects in these models, raising concerns about their safety and potential risk [16]. The ethical considerations may be more prominent, as the face is a key component of an individual's appearance. In contrast, this study proposed that the FA‐TS‐CAT model not only effectively avoids the high‐stimulation and high‐risk factors linked to the face, but also expands the range of test subjects beyond individuals with sensitive skin. The model showed a consistent stimulation and spontaneous recovery trend of the F‐CAT model (Figure 3b).

The compromised barrier function of the stratum corneum, leading to increased permeability, has been recognized as a key mechanism underlying sensitive skin [17]. Studies indicate that the greater number of cell layers of the stratum corneum on the forearms (15 ± 4) than on the face (9 ± 2), potentially accounting for the heightened sensitivity and responsivity of facial skin to external stimuli in comparison to forearm skin [18]. This theory was confirmed in this study that applying the CAT alone to the forearm did not induce significant skin redness or barrier damage as it did on the face (Figure 3d). Heinrich et al. discovered that performing standard tape stripping prior to the patch increases skin sensitivity, and this method is widely employed in contact dermatitis research [19]. Simon and Raj demonstrated a correlation between decrease in the number of stratum corneum layers and compromised barrier function, leading to an enhanced response to capsaicin [6, 20]. Based on these findings, we conducted tape stripping before patching to heighten forearm sensitivity to capsaicin, simulating the facial response in individuals with sensitive skin. During the establishment of the FA‐TS‐CAT model, we observed that the willingness of subjects to participate in the test was inversely correlated with the number of stripping cycles and the duration of patch application (Figure 1), these observations are consistent with Raj and Simon's findings [6, 20]. Tape stripping is a widely used method for skin testing, often employed in evaluating soothing effects or conducting skin omics research [21, 22]. Since it involves mechanical damage, this method can cause discomfort to participants. Therefore, we strictly control the operational procedures to ensure both efficient screening and high tolerance among participants.

Persistent skin redness was observed around the elbows and wrists during practical procedures, possibly due to the dense distribution of blood vessels in these areas and increased vascular reactivity to capsaicin [7]. In addition, overly flexible and too close test areas may interfere with measurements and result in unreliable data. To address these concerns, we devised the experimental areas Z1–Z4, avoiding the elbow and wrist regions. These areas not only delimit specific experimental regions but also provide a degree of adjustment space, making them highly versatile and capable of meeting the testing needs of different individuals. The detection data from Z1–Z4 indicated no significant difference in the four regions of the FA‐TS‐CAT model, with all four samples being measured simultaneously (Table 3). In practical applications, improving the authenticity and reproducibility of the data can be achieved by randomizing samples across these four regions. Therefore, the test efficiency of the FA‐TS‐CAT model was found to be three times higher than that of the F‐CAT model, given that facial models were limited to comparing the split‐face between the control and sample. The conventional double‐layer filter paper patches containing irritants exhibit poor adhesion on the forearm. In contrast, non‐woven double‐layer patches soaked in a 0.01% capsaicin solution and applied directly on the forearm, showed better adhesion and preventing evaporation of the solution. Moreover, the width of the experimental areas was kept consistent with the width of the tape used for stripping (2.5 cm) to ensure that the epidermis was removed uniformly each time. The stability and consistency of the experimental data were significantly ensured by these standardized pre‐control procedures.

To explore the suitability of the FA‐TS‐CAT model for evaluating the efficacy of irritation‐relieving products, an emulsion containing a complex of 4‐*t*‐butylcyclohexanol, *Crocus sativus* flower extract, and *Gentiana rigescens* extract was designed. 4‐*t*‐butylcyclohexanol, a TRPV1 antagonist, has been repeatedly reported for its favorable effects in alleviating the stimulus response induced by TRPV1 activation [23]. *Crocus sativus* flower extract is rich in flavonoids [24], while *Gentiana rigescens* extract contains a large amount of gentiopicroside [25], both of which possess strong anti–inflammatory and analgesic effects. The alleviation of pain and improvement in redness resulting from the combination was found in our internal study. As predicted, excellent relief of pain and redness was observed following the application of the 4‐*t*‐butylcyclohexanol complex in both the F‐CAT and FA‐TS‐CAT models. Redness was observed to improve by 4.92% after using the complex for 10 min, and by 61.18% after 40 min. Meanwhile, volunteers' pain was also significantly relieved (Figure 2). At the same time, a consistent recovery trend after treatments was shown between the F‐CAT and FA‐TS‐CAT groups, indicating broad adaptability.

The FA‐TS‐CAT model not only evaluates the soothing effects on sensitive skin but also has potential for assessing the reparative effects on the barrier function of sensitive skin. In the study, the changes in TEWL caused by the F‐CAT test were weak and uncontrollable, thus insufficient to support an evaluation of the product's effect on skin barrier repair. Conversely, we found that the increase in TEWL induced by the FA‐TS‐CAT model can be used to verify the immediate barrier repair capability of the product, which may be another advantage of this model. Therefore, this forearm model appears to be a valuable tool for evaluating the efficacy of products in improving the skin barrier, warranting further exploration of the optimal operating conditions for this model.

## 5 Conclusion

In this study, we devised an innovative model, the FA‐TS‐CAT, which accurately simulates the natural recovery trend observed in the F‐CAT model and the soothing effect following product application. This model not only enhances the assessment efficiency but also ensures higher safety and adherence of subjects, providing a practical tool for the development and validation of sensitive skincare products and treatments. However, it is important to note that sensitive skin is caused by a variety of complex factors. The model was specifically designed to screen soothing ingredients for subjective tingling sensation and skin redness caused by irritants like capsaicin or lactic acid. Future improvements to this model should aim to expand the stimulation methods to make it more broadly applicable.

## Author Contributions

**Jianhua Zhang:** conception and design, acquisition of data, analysis and interpretation of data, critical revision. **Shichao Liu:** conception and design, project administration, funding acquisition. **Wenjiao Guo:** writing – review and editing, analysis, and interpretation of data. **Na Li:** writing – original draft preparation and interpretation of data. **Yun Huang:** writing – review and editing. All authors have read and agreed to the published version of the manuscript.

## Ethics Statement

This study was conducted in accordance with the Helsinki Declaration and the ICH GCP guidelines as applicable to cosmetic products. The study protocol was approved by the human research ethics committee of EviSkin Testing Technology (Guangzhou) Co., Ltd. (SLLS2023003). All subjects were kept informed of all risks associated with the experiments before signing the informed consent form, and all participated voluntarily in this study. The subjects have agreed to publish the pictures involved in the paper. According to Chinese law, the evaluation of treatment efficacy involving non‐pharmaceutical interventions and non‐disease conditions does not require clinical trial registration.

## Conflicts of Interest

The authors declare no conflicts of interest.

## Data Availability

The data that support the findings of this study are available from the corresponding author upon reasonable request.
